# Advanced adenomas may be a red flag for hereditary cancer syndromes

**DOI:** 10.1186/s13053-020-00164-9

**Published:** 2021-01-12

**Authors:** Swati G. Patel, Heather Hampel, Derek Smith, Dexiang Gao, Myles Cockburn, Fay Kastrinos

**Affiliations:** 1 Department of Internal Medicine, Division of Gastroenterology & Hepatology, University of Colorado Anschutz Medical Campus, 12631 E 17th Avenue, Room 7614, Campus Box 158, Colorado, Aurora 80045 USA; 2 Rocky Mountain Regional Veterans Affairs Medical Center, Aurora, Colorado USA; 3 Division of Human Genetics and the Comprehensive Cancer Center, The Ohio State University, Columbus, OH USA; 4 Department of Pediatrics, Cancer Center Biostatistics Core, University of Colorado Anschutz Medical Campus, Aurora, Colorado USA; 5 University of Colorado Cancer Center, University of Colorado Anschutz Medical Center, Aurora, Colorado USA; 6 Norris Comprehensive Cancer Center, Keck School of Medicine, University of Southern California, Los Angeles, California USA; 7 Division of Gastroenterology & Hepatology, Columbia University Medical Center, New York, NY USA

**Keywords:** Colorectal cancer, Colorectal polyps, Adenomas, Genetic testing, Lynch syndrome

## Abstract

**Background:**

16–25% of colorectal cancers (CRCs) diagnosed under age 50 are associated with hereditary cancer syndromes. Advanced adenomas are considered precursors to CRC. Although polyp removal prevents cancer, polypectomy does not change underlying genetic risk. Patients with isolated advanced polyps do not currently qualify for genetic testing unless they have a personal or family history of cancer.

**Aim:**

Describe the prevalence of hereditary cancer syndromes among patients with advanced colorectal polyps.

**Methods:**

We performed a single center retrospective review from 2015 to 2019 of patients who underwent germline genetic testing with indication for testing listed as colorectal polyp. We excluded patients with a personal history of CRC and those with ≥10 cumulative polyps. We collected patient demographics, polyp characteristics, family history data and genetic testing results from the medical record. Discrete variables were reported as frequency and percentages and continuous variables reported as mean with range.

**Results:**

A total of 42 patients underwent genetic testing due to a personal history of advanced adenoma. 17% of patients met current genetic testing criteria. All patients underwent multi-gene panel testing. Two patients (4.8%) had a germline pathogenic mutation (one in *MLH1* and one in *CHEK2*). The patient with an *MLH1* mutation met current criteria for genetic testing (PREMM5 score 5.8), however the patient with the *CHEK2* mutation did not. Both mutation carriers had a personal history of synchronous or metachronous advanced adenomas. 38% had a variant of uncertain significance.

**Conclusions:**

5% of patients with advanced adenomas in our retrospective series had a pathogenic germline mutation in a cancer predisposition gene. Though the patient with a pathogenic mutation in *MLH1* met current clinical criteria for genetic testing, this was not recognized prior to referral; he was referred based on a personal history of advanced adenoma. Advanced polyps may be a red flag to identify patients who are at risk for hereditary cancer syndromes.

## Introduction

Up to 10% [1] of all colorectal cancers (CRCs) and 16% [2] of CRCs diagnosed under the age of 50 are associated with a germline pathogenic variant in a cancer predisposition gene. It is extremely important to identify these patients because they are at risk of multiple cancers, often at younger ages than the general population [3]. There are effective, guideline-based cancer risk reduction strategies including endoscopic screening, chemoprevention and prophylactic surgery [3]. Current efforts to identify hereditary cancer syndrome patients rely on personal or family history of cancer. Despite these efforts, hereditary syndromes are grossly under-recognized. For example, less than 5% of Lynch Syndrome carriers, the most common hereditary CRC syndrome, are aware of their diagnosis [4].

Colonoscopy is the most common CRC screening test in the United States [5] and is considered a cancer prevention test because pre-cancerous colorectal polyps, such as adenomas, can be detected and removed [6]. Colonoscopy use is increasing in all age groups in the United States for a variety of indications including screening, family history of colorectal neoplasia, polyp and CRC surveillance and evaluation of symptoms [7]. Though increased use of colonoscopy and polyp removal are great successes in cancer prevention, interrupting the natural history to cancer does not change the patient’s underlying genetic diathesis.

Prior efforts to target adenomatous polyps as red flags for hereditary syndromes, such as Lynch Syndrome, have focused on tissue-based screening methods used for CRC which include immunohistochemistry (IHC) testing for mismatch repair (MMR) protein expression or polymerase chain reaction (PCR) for microsatellite instability (MSI) markers. Disappointingly, these screening methods in colorectal adenomas have only shown a 50–70% sensitivity for identifying germline Lynch Syndrome mutation carriers [8] and do not screen for the other hereditary syndromes. This limited test sensitivity coupled with the cost and logistic challenges of implementing tumor based screening [9] have been barriers to using colorectal adenomas to identify hereditary syndrome patients.

With decreased cost and increased access to germline testing [10], targeting patients with advanced adenomas for direct germline genetic testing may be a direct and affordable way to identify hereditary cancer syndrome patients before they develop cancer. The aim of our study was to describe the yield of germline genetic testing results in patients with advanced adenomas.

## Materials & methods

### Participants

This was a single-center retrospective study conducted at a tertiary academic medical center. Our hereditary cancer clinic sees patients referred state-wide and includes an in-person as well as telehealth practice. Eligible participants were identified from our institution’s cancer genetics database (Progeny®) from June 2015–October 2019. This database includes all patients who have undergone genetic counseling and family pedigree construction. For those patients who proceeded with germline genetic testing, genetic testing results are also recorded in this database.

Inclusion criteria for this study were: (1) age over 18, (2) personal history of colorectal adenoma as the primary indication for referral to hereditary cancer clinic and (3) at least one advanced adenoma. Patients were excluded from our analysis if there was a known genetic condition within the family or the patient had a personal history of ≥10 colon polyps (met polyposis genetic testing criteria).

### Definitions & Outcomes

By convention [6], advanced adenoma was defined as a polyp in the colon or rectum with one of the following features: (1) ≥ 1 cm as documented by the endoscopist, (2) with villous architecture on histology or (3) with high-grade dysplasia.

In accordance with the American College of Medical Genetics, a pathogenic variant was defined as alterations with sufficient evidence to classify as capable of causing disease. A likely pathogenic variant was defined as alterations with strong evidence in favor of causing disease. A variant of uncertain significance was defined as alterations with limited and/or conflicting evidence regarding pathogenicity. Likely benign and benign variants were defined as alterations with strong evidence against pathogenicity.

The primary outcome of our study was a pathogenic or likely pathogenic germline variant.

### Data collection

Per routine clinical care for patients seen in our hereditary cancer clinic, a comprehensive medical and cancer history was obtained for each participant. A three generation cancer family history was collected on all participants and a pedigree was also constructed. Based on this medical record documentation, we determined whether participants met clinical criteria for genetic testing [3], including a first-degree relative diagnosed with CRC under age 50 [3], Amsterdam II criteria [11] (3 relatives with a Lynch Associated Cancer, 2 consecutive generations, 1 cancer diagnosis under age 50) or a PREMM5 score ≥ 5% (risk prediction model that takes into account personal and family history of cancer) [12].

Patients who proceeded with genetic testing underwent germline DNA sequencing using blood or saliva by a Clinical Laboratory Improvement Amendments (CLIA) approved laboratory. Since genetic testing was part of clinical care, the specific company used for genetic testing and the genes selected for testing were variable; information of specific genes and/or multi-gene panels was collected for each participant. We searched for each variant in ClinVar, a database hosted by the National Institutes of Health which aggregates information about genomic variants, to determine whether there were any updates in classification of variants.

Colonoscopy indication, polyp size, polyp location and polyp histology were collected from the medical record. If available, IHC for MMR proteins was extracted from the pathology records. Documentation of family history was extracted from clinical progress notes up to one year prior to the colonoscopy and the colonoscopy procedure report, if listed as an indication for the procedure.

### Data Management & Statistical Analysis

Data was extracted from Progeny® and our institution’s electronic medical record (Epic®) and entered into a database designed in REDCap (Research Electronic Data Capture) hosted at the University of Colorado. REDCap is a secure, HIPAA-compliant, web-based application designed to support data capture for research studies.

Cohort characteristics were summarized using frequency counts and percentages for categorical variables and means with standard deviation for continuous variables. Statistical analysis was performed using SAS Version 9.4 (SAS Institute, Cary, NC). This study was approved by the Colorado Multiple Institutional Review Board.

## Results

### Baseline characteristics

A total of 42 participants met our inclusion criteria. The mean age of patients was 44.7 years; 45.2% (*n* = 19) were male (Table 1). One patient had a personal history of cancer (basal cell skin cancer), 11.9% (*n* = 5) of patients had a personal history of a synchronous or metachronous advanced adenoma and 23.8% (*n* = 10) reported a first-degree relative with CRC.

**Table 1:** Patient, polyp and family history characteristics

	*N* = 42
Mean age at polyp diagnosis, years	44.6 (range 23–57)
Sex, n (%)	
Male	19 (45.2)
Female	23 (54.8)
Personal History of Synchronous/Metachronous AA	
Yes	5 (11.9)
No	37 (88.1)
FDR with CRC, n (%)	
Yes	10 (23.8)
No	32 (76.2)
Colonoscopy Indication	
Symptoms	20 (47.6)
Screening for FH of CRC	7 (16.7)
Average Risk Screening	14 (33.3)
Polyp Surveillance	1 (2.4)
Polyp Size, n (%)	
< 10 mm	2 (4.8)
10–20 mm	21 (50.0)
21–30 mm	16 (38.1)
31–40 mm	2 (4.7)
> 40 mm	1 (2.4)
Polyp Histology, n (%)	
Tubular adenoma	39 (92.9)
Tubulovillous adenoma	2 (4.8)
Villous adenoma	1 (2.4)
Immunohistochemistry Results	
Not performed	29 (69.0)
Normal	12 (28.6)
Absence of MLH1/PMS2	1 (2.4)
Meets Amsterdam II Criteria, n (%)	
Yes	3 (7.1)
No	39 (92.9)
PREMM5 Score, n (%)	
≥ 5	6 (14.3)
≥ 2.5	14 (33.3)
Unable to calculate*	15 (35.7)

*FH* Family History, *CRC* colorectal cancer, *FDR* First Degree Relative, *CRC* Colorectal Cancer, *PREMM5* Lynch syndrome prediction model

^a^PREMM5 can only be calculated if there is a personal or family history of cancer. A score of ≥5 meets NCCN criteria for genetic testing. Recent studies have shown increased sensitivity when PREMM5 score is ≥2.5, though this does not meet current NCCN criteria

### Colonoscopy and polyp findings

The documented indications for colonoscopy were symptoms (*n* = 20, 48%), family history of CRC (*n* = 7, 17%), screening (*n* = 14, 33%) and polyp surveillance (n = 1, 2%). The majority of advanced adenomas were 10–30 mm (*n* = 37, 88%) and tubular adenomas (*n* = 39, 93%). One patient had abnormal immunohistochemistry (IHC) with loss of *MLH1*/*PMS2*, with reflex testing that showed *braf-*wild type. The majority did not have IHC performed (*n* = 29, 69%) and the rest showed normal protein staining (*n* = 12, 29%).

### Germline genetic testing criteria and results

7.1% (*n* = 3) patients met Amsterdam II criteria, 14.3% (*n* = 6) of patients had a PREMM5 score of ≥5% and two participants met both criteria. Thus, 16.7% (*n* = 7) of our cohort met guideline-based genetic testing criteria. Of the seven participants who met clinical criteria for genetic testing, only two had any family history documented in the medical record (both as indications for the colonoscopy) and neither of these two participants listed family history in the referral placed to the hereditary cancer center.

**Fig. 1:**
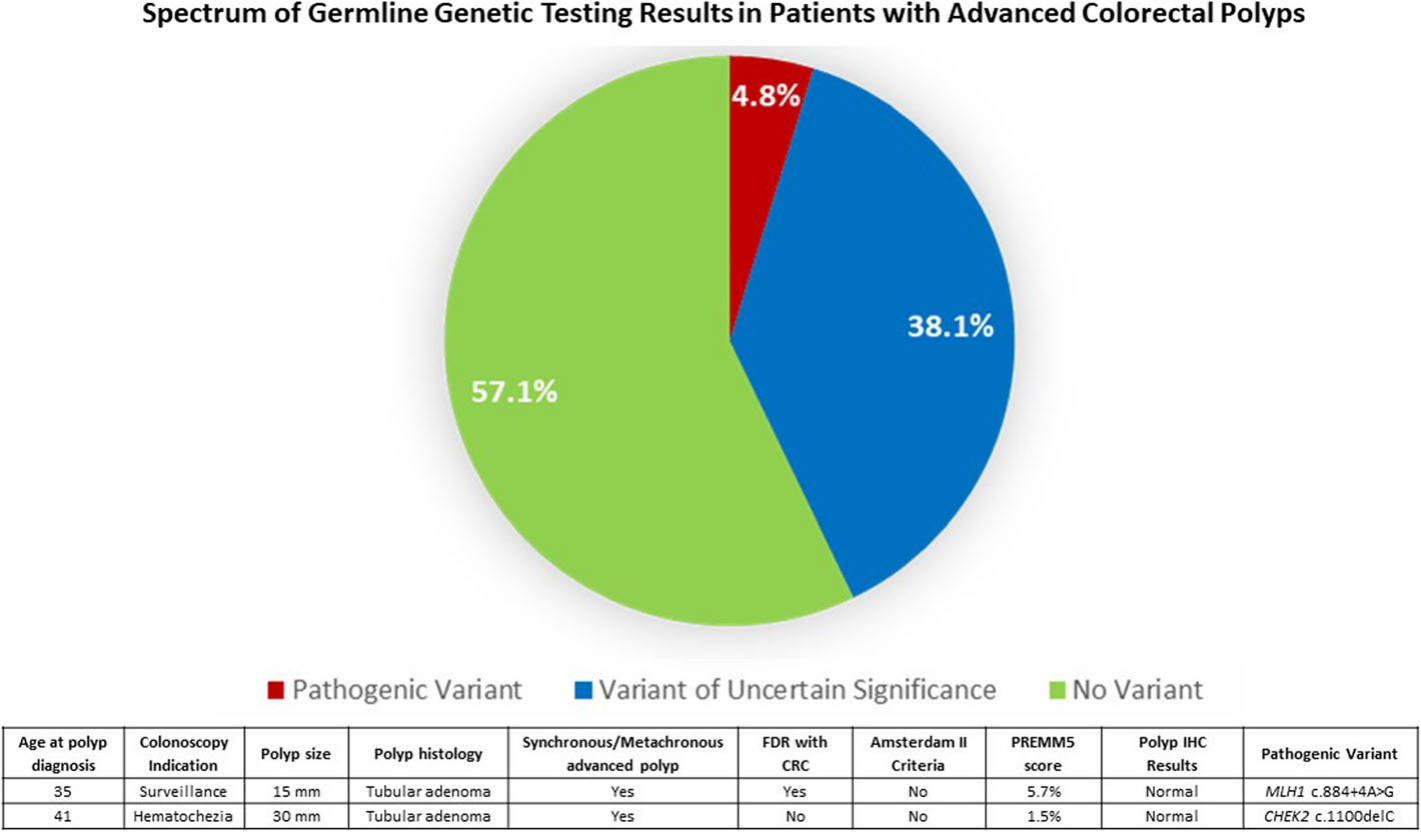
Spectrum of germline genetic testing results in patients with advanced colorectal polyps. FDR: first-degree relative; IHC: immunohistochemistry

Two patients (4.8%) had a pathogenic germline variant, one in *MLH1* (c.884 + 4A > G) and one in *CHEK2* (c.1100delC) (Fig. 1). The patient with *MLH1*-Lynch Syndrome underwent his first colonoscopy at 32 years for evaluation of hematochezia were an 18 mm rectal adenoma was removed. Surveillance colonoscopy at the recommended 3-year interval (age of 35) revealed a metachronous 15 mm adenoma with high-grade dysplasia in the transverse colon. This patient met genetic testing criteria based on his family history (first-degree relative with early-onset CRC and PREMM5 score > 5%). Family history was not documented as an indication for his colonoscopies nor included in clinical notes. The reason for referral to hereditary cancer clinic was his personal history of advanced adenoma at a young age. IHC of his polyp tissue was normal. He was the first member of his family identified to have Lynch Syndrome.

The patient with a *CHEK2* mutation underwent his first colonoscopy at age 40 due to hematochezia where he had three tubular adenomas removed (12, 15 and 30 mm). He had a second degree relative with CRC and did not meet genetic testing criteria (PREMM5 score was 1.5%). IHC of his polyp tissue was also normal.

38.1% (*n* = 16) participants had a variant of uncertain significance (VUS); 11 VUS’ in genes strongly associated with a risk of CRC. 57.1% (*n* = 24) had no variant reported (Table 2). The single patient who had loss of *MLH1*/*PMS2* had a 47-gene panel and no variant was found.

**Table 2:** Germline variants identified

Number of genes tested	Gene	Variant	Classification
47	*MSH3*	c.2180G > A	VUS
83	*CDH1*	c.394G > A	VUS
47	*MSH6*	c.1211A > G	VUS
42	*MLH1*	c.884 + 4A > G	Pathogenic
83	*BLM*	c.968A > G	VUS
47	*RAD51*	c.878C > T	VUS
47	*CHEK2*	c.1100delC	Pathogenic
47	*CDKN2A*	c.407dup	VUS
46	*POLE*	c.5744 T > C	VUS
7	*MUTYH*	c.700G > A	VUS
47	*APC*	c.-30,478 T > C	VUS
83	*POLE*	c.3881G > T	VUS
47	*APC*	c.423-3_423-2del	VUS
47	*MSH6*	c.2979A > G	VUS
29	*CHEK2*	del exon 13	VUS
47	*APC*	c.5140G > A	VUS
29	*POLD1*	c.1040C > T	VUS
29	*TP53*	c.814G > T	VUS

*VUS* Variant of Uncertain Significance. Gray shading indicates genes associated with an increased risk of CRC

## Discussion

Approximately 5% of those with advanced adenomatous colorectal polyps in our series had a pathogenic germline variant associated with increased CRC risk. These findings re-invigorate the concept that advanced adenomas can serve as red flags to identify those who may be at risk for hereditary cancer syndromes.

Our results are consistent with prior work [8] showing that tissue based-screening of adenomas is not a sufficiently sensitive approach given that neither of the two participants with pathogenic germline variants had abnormal tissue screening. The one patient who had absence of *MLH1*/*PMS2*, had follow-up *braf* testing consistent with sporadic tumor and did not have any variant identified on comprehensive (47-gene panel) germline testing. It is unclear if the limited sensitivity for tissue-based screening is due to when the MMR defect occurs along the adenoma-carcinoma sequence or variable MMR-phenotypes depending on genotype [8]. IHC of the *MLH1-*Lynch Syndrome patient was normal. Our results show that directly assessing the germline is a potential way to bypass the limitations of tissue-based screening.

It is important to note that although 16.7% (*n* = 7) of our cohort met family history clinical criteria for germline genetic testing, this family history was not documented in the medical record for five of these patients prior to genetic counseling and pedigree construction. For the two patients where family history of CRC was documented (both as the indication for the colonoscopy), family history was not the listed reason for referral to hereditary cancer clinic. These results are consistent with prior work showing that clinical documentation of cancer family history is poor [13, 14] and even when family history is documented, it is incomplete and there is poor recognition of patients who meet genetic testing criteria [15]. Although the Lynch Syndrome patient we identified met established criteria for genetic testing based on his family history, these criteria were not identified or acted upon prior to his advanced adenoma diagnosis. Similar to our approach to universal screening for hereditary syndromes in CRC patients [16], advanced adenomas would circumvent challenges in family history collection and action.

While we cannot conclude causality between the germline variants identified and the presence of advanced colorectal polyps, it seems plausible that the advanced adenomas detected in our *MLH1*-Lynch patient are driven by the germline defect. Similarly, we cannot presume that the *CHEK2* variant caused the three advanced adenomas found in our second patient. Regardless of whether the germline defect caused the advanced polyps, identification of these variants and implementation of cancer risk reduction measures (screening, chemoprevention, prophylactic surgery) can have a significant impact on the proband and at-risk family members.

There are several limitations to our analysis. Our sample size is small and there is inherent selection and referral bias in the retrospective design of our study. Furthermore, the number of genes tested was not standardized since it was part of routine clinical care, however given that all participants underwent testing after 2015, the majority had multi-gene panels (Table 2). Larger, prospective studies are needed to confirm our results and assess the precise yield and spectrum of germline variants in unselected patients with advanced adenomas.

Despite these limitations, our study results suggest that direct germline evaluation of patients with advanced adenomas can help identify hereditary syndromes. If confirmed in larger studies, incorporating a personal history of advanced adenomas in genetic testing criteria along with age and family history of cancer can potentially broaden the current paradigm for genetics evaluation. Future directions of this work include identifying risk factors for harboring germline pathogenic variants, such as age at diagnosis and family history of cancer, among patients with advanced adenomas to help inform how to incorporate advanced polyp findings into existing hereditary risk assessment tools.

## Conclusions

Approximately 5% (2/42) of those with advanced adenomas in our series had a pathogenic germline variant in a cancer predisposition gene. These findings re-invigorate the concept that advanced adenomas can serve as red flags to identify those who may be at risk for hereditary cancer syndromes. As the uptake of colonoscopy and polypectomy are increasing in all age groups, including those < 50 years, understanding the prevalence and spectrum of germline variants among patients with pre-cancer, as we currently do with CRC, will be increasingly important in the identification of high-risk individuals and their family members.

## Data Availability

Not applicable.

## Abbreviations

CRC: Colorectal Cancer
NCCN: National Comprehensive Cancer Network
IHC: Immunohistochemistry
MMR: Mismatch Repair
MSI: Microsatellite Instability
PCR: Polymerase Chain Reaction
